# PTBP1 protects Y RNA from cleavage leading to its apoptosis-specific degradation

**DOI:** 10.1038/s41420-024-02080-6

**Published:** 2024-07-12

**Authors:** Takeshi Kamakura, Kazuaki Kameda, Masahiko Manabe, Kan Torii, Yuki Sugiura, Seiko Ito, Shunya Nakayama, Takanobu Shimizu, Etsuko Nagashima, Kosuke Kamiya, Masahiro Oka, Masafumi Tanaka, Motoyuki Otsuka, Masato Ohtsuka, Ai Kotani

**Affiliations:** 1 Department of Regulation of Infectious Cancer, Research Institute of Microbiological Disease, Osaka University, Suita, Osaka 565-0871 Japan; 2 Department of Innovative Medical Science, Tokai University School of Medicine, Isehara, Kanagawa 259-1193 Japan; 3 Department of Immunobiology, Yale University School of Medicine, New Haven, CT 06519 USA; 4 Multi-Omics Platform, Center for Cancer Immunotherapy and Immunobiology, Kyoto University, Kyoto, 606-8501 Japan; 5 Laboratory of Veterinary Physiology, College of Bioresource Sciences, Nihon University, Fujisawa, Kanagawa 252-0880 Japan; 6 Department of Molecular Life Science, Division of Basic Medical Science and Molecular Medicine, Tokai University School of Medicine, Isehara, Kanagawa 259-1193 Japan; 7 Department of Gastroenterology and Hepatology, Academic Field of Medicine, Density and Pharmaceutical Sciences, Okayama University, Okayama, 700-8558 Japan

**Keywords:** RNA, Apoptosis, Proteasome

## Abstract

Some RNAs such as 28S rRNA, U1 small nuclear RNA (snRNA), and Y RNAs are known to be cleaved during apoptosis. The underlying mechanism, functions, and biological significance of RNA degradation in apoptosis remain elusive. Y RNAs are non-coding RNAs widely conserved from bacteria to mammals, and are major components of Ro ribonucleoprotein (RNP) complexes which contain the 60 kDa Ro protein (SS-A) and the 50 kDa La protein (SS-B). The autoantigenic Ro and La proteins were identified by autoantibodies present in the sera from patients with Systemic lupus erythematosus (SLE) and Sjögren’s syndrome (SjS). We previously identified novel, functional small RNAs named AGO-taxis small RNAs (ASRs) that are specifically bound to Argonaute protein 1 (AGO1), which are processed from Y RNAs. Cell-free analysis combined with fractionation methods revealed that the apoptosis-specific biogenesis of ASRs or cleavage of Y RNA was induced by truncation of polypyrimidine tract-binding protein 1 (PTBP1), which is an endoribonuclease inhibitor of Y RNAs by caspase 3. Caspase 3-resistant PTBP1 mutant protected cleavage of Y RNAs in apoptosis induced by staurosporine. Furthermore, caspase 3-resistant PTBP1 mutant knock-in mice showed elevated cytokines, dysregulation of the germinal center formation compared to the wild-type mice at LPS stimulation, and high positivity of antinuclear antibody. Those results suggest that cleavage of Y RNAs or biogenesis of ASR during apoptosis has critical biological functions and their deregulation result in immune dysregulation and the formation of autoantibody, possibly leading to the development of autoimmune diseases.

## Introduction

Apoptosis is essential for the development and homeostasis of metazoans [1]. In an apoptotic cell, some components are decomposed by their own proteases or nucleases [2, 3]. While the processes associated with chromatin fragmentation, cytoskeleton disruption, nucleus condensation, and membrane blebbing are extensively studied and well understood, the mechanisms and functions of apoptotic RNA degradation remain unelucidated [4, 5].

Active RNA cleavage in apoptotic cells is observed for a few non-coding RNA species such as 28S rRNA, U1 small nuclear RNA (snRNA), and Y RNAs. 28S rRNA, a part of the large subunit of the ribosome, is transcribed by RNA polymerase I in the nucleolus. While 18S rRNA remains intact during apoptosis, 28S rRNA is immediately fragmented [6]. RNA forms the U1 snRNP complex, which is involved in mRNA splicing. It is also cleaved in apoptotic cells [7]. During apoptosis, one of the components, U1-70K, is cleaved by the activated caspase 3 and the single-stranded 5′-end is simultaneously truncated by an unknown ribonuclease [8]. Y RNAs undergo rapid cleavage by caspase 3 during apoptosis [9]. Y RNAs are non-coding RNAs that are widely conserved from bacteria to mammals [10]. Humans have four Y RNAs: Y1, Y3, Y4, and Y5 RNA. In the nucleus, Y RNAs contribute to DNA replication initiation through formation of the replication origin [11, 12]. Y RNAs form Ro-RNP complex with Ro60, which has been reported to be one of the common autoantigens observed in autoimmune diseases, to regulate the cytoplasmic localization of Ro protein by masking the nuclear localization signal of the protein [13]. Over 20 years ago, Y RNA cleavage in human apoptotic cells were observed. However, understanding the mechanisms and identifying the responsible ribonuclease has been challenging. This difficulty arises in part from the heterogeneous sizes of the Y RNA cleavage/degradation products. This heterogeneity suggests that the cleavages don’t stem from a single endonucleolytic reaction. Instead, multiple endonucleolytic cleavages or a mix of endo- and exonuclease activities might be needed to produce these varied products [14].

Autoantibodies against these ribonucleocomplexes (ribosome, U1 snRNP, and Ro-RNP) are often detected in the sera of patients with systemic autoimmune diseases. This is indicative of the involvement of apoptotic RNA cleavage in these pathological conditions [15–18].

The study of small RNA, including microRNA (miRNA), endo-small interfering RNA (siRNA), and PIWI-interacting RNA (piRNA), has greatly expanded after the emergence of next-generation sequencing [19–21]. We previously identified a new class of small RNAs that specifically bind to Argonaute protein 1 (AGO1), but not AGO2, prepared from Epstein-Barr virus (EBV)-positive lymphoma cells, by small RNA sequencing [22]. These small RNAs named AGO-taxis small RNAs (ASRs) exhibited RNA interference activity and dramatically increased during the EBV lytic infection, which was accompanied with massive cell death.

To investigate the correlation and biological significance of this small RNA cleavage, apoptosis, and pathogenesis of autoimmune diseases, it is crucial to elucidate the detailed mechanism of RNA cleavage in apoptosis.

Here, we conducted a cell-free assay in combination with some fractionation methods to reveal that Y RNA cleavage in apoptosis is caused by truncation of polypyrimidine tract-binding protein 1 (PTBP1) which is a well-known splicing factor [23] by caspase 3. Caspase 3-resistant PTBP1 knock-in mice (*Ptbp1*^*mut/mut*^ mice) showed elevated cytokines in the serum nonetheless low germinal center formation in the spleen at LPS stimulation and high positivity of antinuclear antibody, which suggests the dysregulation of Y RNA cleavage in apoptosis is involved in immune dysregulation, possibly resulting in formation of autoantibody.

## Results

### Y RNA cleavage is activated in apoptotic cells

**Fig. 1:**
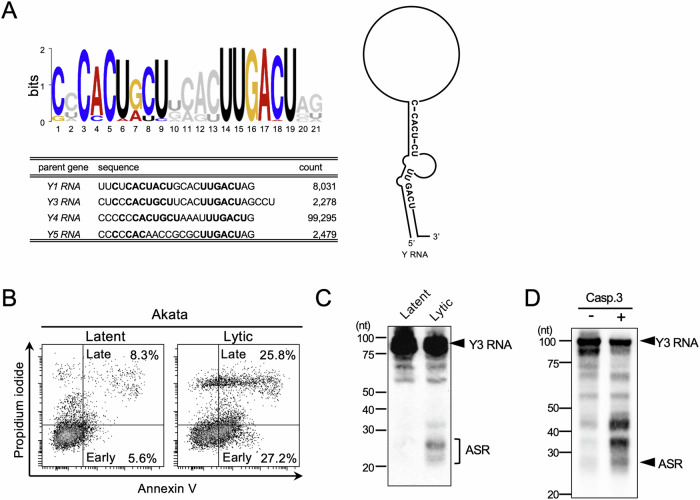
Y RNA cleavage is activated in apoptotic cells. **A** Common motif of ASRs was identified by MEME motif search. The motif, indicated in the capital letter, was plotted on Y RNA common structure. Sequences of the most abundant ASRs from each Y RNA were analyzed. **B** EBV lytic infection was induced by BCR stimulation using anti-human IgG antibody. After 24 h, apoptosis induction was investigated by flow cytometry. Annexin V single positivity shows early apoptotic cells and double positivity shows late apoptotic cells. **C** Y3 RNA and ASRs cleavage product were detected by northern blotting for latent or lytic infection of EBV (**C**), and with or without caspase 3 (**D**). Similar results were obtained in two independent experiments.

The consensus motif of the small RNAs, “ASRs” was UUGACU [5], known to be conserved in the stem region of the non-coding Y RNA, was identified as the binding motif of Ro autoantigen. A common structure of Y RNA was drawn using secondary structure of Y RNA [24]. The small RNAs were found to be derived from the 3′ stem regions of Y RNA via cleavage of the single-strand loop region (Fig. 1A).

The elevation in ASRs levels along with EBV reactivation suggests that Y RNA cleavage depends on cell death, which massively occurs during EBV lytic infection. Apoptotic cells were defined as Annexin V-positive cells [25]. After 24 h of B cell receptor (BCR)-mediated lytic cycle induction, the number of apoptotic cells was increased in Akata cells (Annexin V^+^PI^−^; 27.2%, Annexin V^+^PI^+^; 25.8%, total Annexin V^+^; 53.0%) as compared to cells without induction (Annexin V^+^PI^−^; 5.6%, Annexin V^+^PI^+^; 8.3%, total Annexin V^+^; 13.9%) (Fig. 1B). Under the condition, Y RNA cleavage was observed by northern blotting (Fig. 1C). Additionally, Y RNA cleavage was induced by activated caspase 3 in the cell-free system in vitro (Fig. 1D). These results indicate that Y RNA cleavage is activated in apoptotic cells and is coordinated by caspase 3, as shown before [9].

### Y RNA cleavage is independent of Drosha, Dicer, and RNase L

**Fig. 2:**
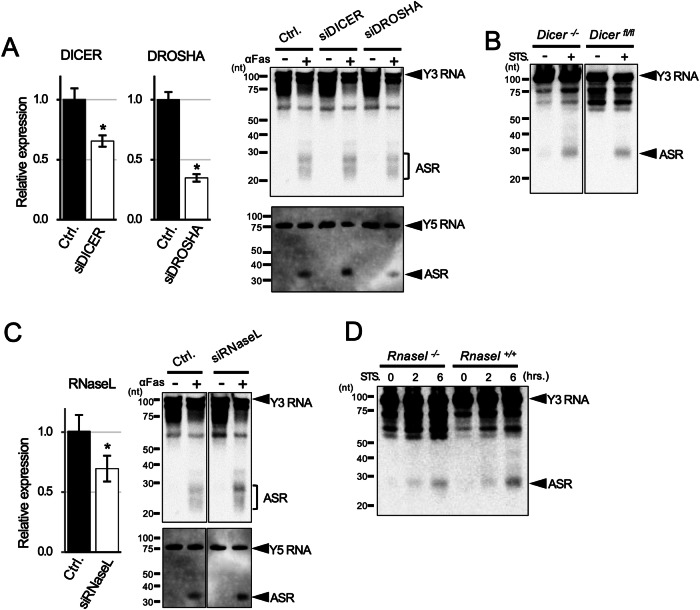
Y RNA processing is independent of DROSHA, DICER, and RNase L. **A** Expression of DROSHA and DICER in the Jurkat cells transfected with control siRNA (Ctrl.), siDROSHA, or siDICER by real-time PCR (*n* = 3) normalized by GAPDH (Left). Expression of Y RNA fragments in those cells, where apoptosis was induced by anti-Fas antibody (clone: CH-11), by northern blotting (Right). Upper panel, Y3 RNA and ASR; lower panel, Y5 RNA and ASR. **B** Dicer-knockout or wild-type (floxed Dicer) MEFs were cultivated with or without 10 µM staurosporine for 2 h, followed by the detection of Y3 RNA cleavage by northern blotting. **C** Expression of RNase L in the Jurkat cells transfected with control siRNA (Ctrl.) or siRNase L by real-time PCR (*n* = 3) normalized by GAPDH. Y3 RNA or Y5 RNA cleavage was also detected by northern blotting as in (**A**). **D** Rnasel-knockout or wild-type MEFs were stimulated with 10 μM staurosporine for 2 or 6 h, followed by the detection of Y3 RNA cleavage by northern blotting. Similar results were obtained in two independent experiments. Student’s *t*-test was used for the statistical analysis. *P < 0.05, error bars; mean ± s.d.

ASRs are processed independently from Drosha [5]. Then we examined whether ASRs were processed by Dicer. To study the involvement of Dicer and Drosha in ASRs processing, that is Y RNA cleavage, the expression of ASRs in apoptosis-inducing cells was measured by the siRNA-mediated knockdown of the genes. As a result, Y cleavage was almost equally observed in both Dicer- or Drosha-knockdown or control cells (Fig. 2A). No reduction of the processing was observed. Furthermore, the results were confirmed in Dicer-deficient MEF cells following apoptotic induction by staurosporine (Fig. 2B). These results indicate that Y RNAs are neither cleaved by Dicer nor Drosha.

Several viral single-strand RNAs are known to be cleaved by RNase L [26], which is activated during apoptosis [27]. We examined whether Y RNA was cleaved by RNase L. The expression of ASRs in apoptosis-inducing cells was measured by the siRNA-mediated knockdown of RNase L. As a result, Y RNA cleavage was almost equally observed in RNase L-knockdown and control cells (Fig. 2C). Furthermore, the results were confirmed in Rnase L-deficient MEF cells following apoptotic induction by staurosporine (Fig. 2D). Again, no reduction of the processing was observed. Thus, the degradation of Y-RNA occurred without Dicer or RNase-L, which suggest that other responsible molecules are involved in this process.

### Caspase 3 is involved in apoptosis-dependent Y RNA cleavage

**Fig. 3:**
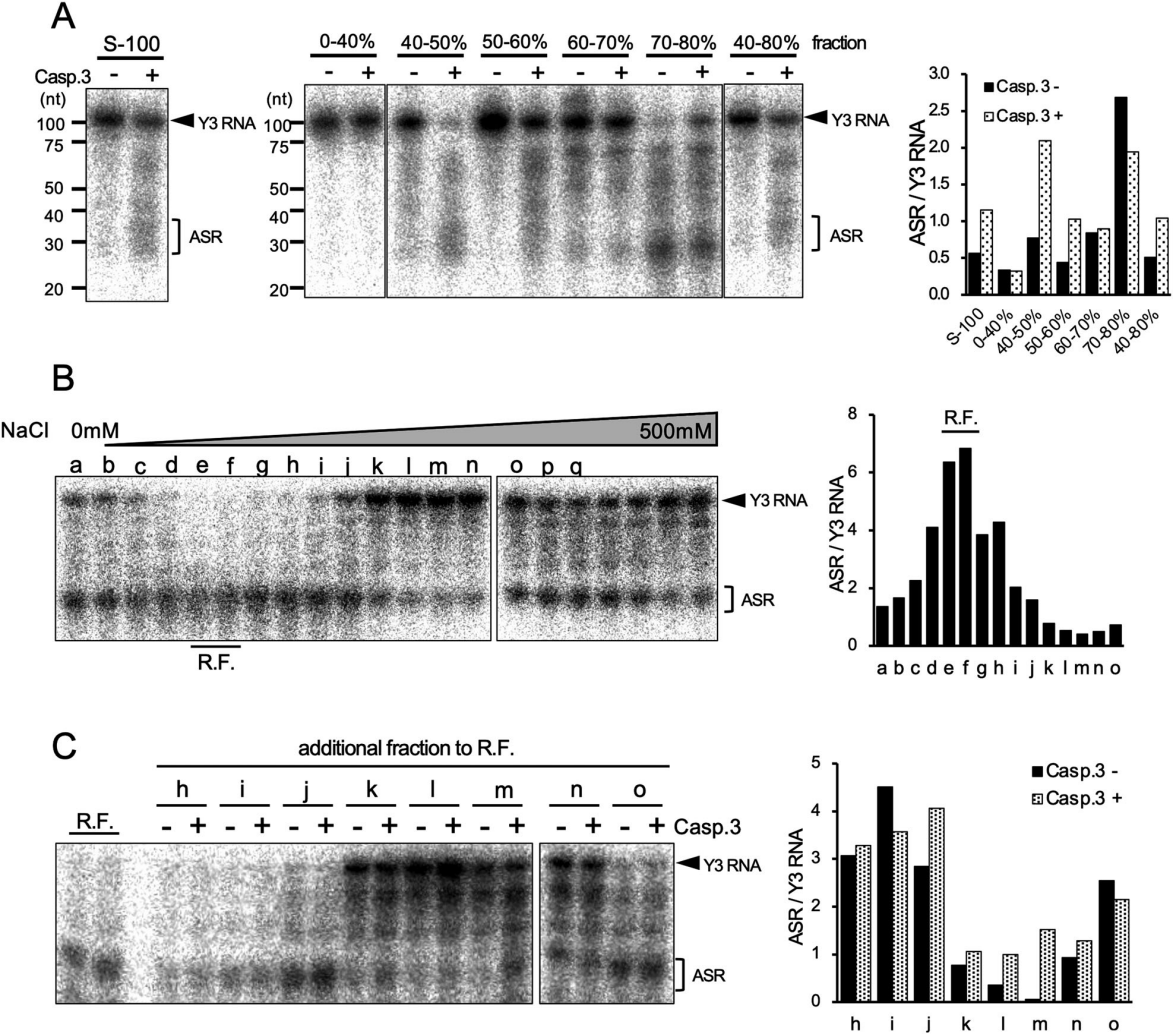
Y RNA processing involves ribonuclease and its inhibitor, responsible for caspase 3 dependency. **A** S-100 fractions separated by ammonium sulfate fractionation (left). The ratio of ASR/Y3 RNA was calculated and plotted in a bar graph (right). **B** Ammonium sulfate fraction (50–60%) separated by IEC. RNase activity was detected in fractions e and f, which were defined as RNase fraction (R.F.) (left). The ratio of ASR/Y3 RNA was calculated and plotted in a bar graph (right). **C** RNase activity in the R.F.re mixed with other fractions with or without caspase 3 (left). The ratio of ASR/Y3 RNA was calculated and plotted in a bar graph (right). Similar results were obtained in two independent experiments.

To identify the molecules responsible for Y RNA cleavage, the S-100 fraction from Jurkat cells was fractionated using saturated ammonium sulfate (SAS). Each fraction was treated with the activated caspase 3, and then Y RNA processing was examined. As shown in Fig. 3A, while no ASRs were observed in 0-40% fraction, some production of ASRs are observed in 40–50%, 50–60%, 60–70%, and 70–80% fraction. Production of ASRs did not depend on caspase 3 in 60–70% and 70–80% fraction. Among 40–50% and 50–60% fraction which showed caspase 3-dependent production of ASRs, 50–60% fraction which showed a 2.3-fold increase in caspase 3-dependent Y RNA cleavage activity, calculated as ASRs/Y3 RNA, as compared with that in the control without caspase 3 (1.03 versus 0.44) (Fig. 3A) was further separated by IEC. For the separated fractions, the cleavage activities were examined with or without caspase 3. We failed to observe the dependency of caspase 3 for Y RNA processing in all fractions. The processing was examined in the reaction without caspase 3 (Fig. 3B). Fractions e and f showed 6.36 and 6.84 processing activities, which were 2.56- and 2.75-fold higher, respectively, than the average in this assay (2.48). These fractions showed high RNase activity and were defined as RNase fraction (R.F.). Based on the results, it was hypothesized that molecules determining caspase 3 dependency comprised R.F. and other fractions, which may contain the inhibitory activity of RNase. To test this hypothesis, R.F. was mixed with other IEC fractions and incubated with the activated caspase 3 (Fig. 3C). We detected 0.77, 0.36, 0.06, and 0.93 processing activities without caspase 3 with k to n fractions, which corresponded to 3.4, 7.4, 45.7, and 2.8-fold reduction, respectively, as compared to the average processing activity of R.F (2.6). Caspase 3-dependent processing was recovered upon mixing the fraction from k to m (Fig. 3C). The processing activity of fraction m without and with caspase 3 was 0.06 and 1.52, respectively, indicating that a 26.4-fold increase in the processing activity was observed in the reaction with caspase 3 as compared to the reaction without caspase 3. These results suggest that RNase activity is inhibited by one or several factors from IEC fractions k to m and that the inhibition activity was deactivated by caspase 3.

### Caspase 3-resistant PTBP1 protects Y RNA degradation from RNase

**Fig. 4:**
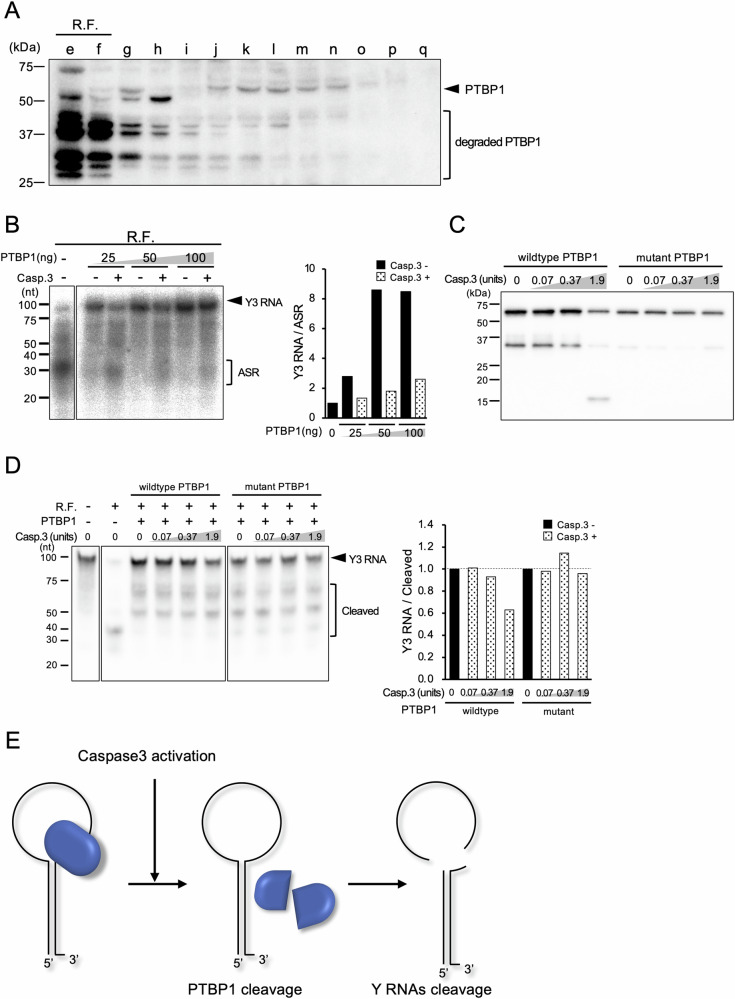
PTBP1 is responsible for the caspase 3-dependent Y RNA cleavage. **A** Expression of PTBP1 in an equal volume of each IEC fraction analyzed by western blotting with anti-PTBP1 antibody. **B** Y3 RNA cleavage with or without recombinant PTBP1 (left). Y3 RNA/ASR ratios were calculated and normalized by the reaction without PTBP1 and plotted as a bar graph (right). **C** Cleavage of WT or caspase 3-resistant PTBP1 by caspase 3 by western blotting. **D** Y3 RNA cleavage detected by northern blotting under the condition of (C) (left). The cleavage activities were calculated as a ratio of Y3 RNA/cleaved products, then normalized by the reaction without caspase 3, respectively (right). **E** Schematic representation of the caspase 3-dependent Y RNA cleavage in apoptosis. Similar results were obtained in two independent experiments.

We thought that the RNase inhibitor was truncated by caspase 3. Several proteins were shown to be bound to Y RNA [28]. One of these proteins is PTBP1 (also known as heterogenous nuclear ribonucleoprotein I: hnRNP I), which binds to the loop region of Y RNA where it is cleaved during the biogenesis of ASRs [29]. Furthermore, the cleavage of PTBP1 by caspase 3 was reported, but its significance has been undetermined [30]. IEC fractions were analyzed by Western blotting with anti-PTBP1 antibody, and significant band densities were mainly detected in fractions with inhibitory activity for cleavage, while degradation of PTBP1 was detected in R.F. (Fig. 4A). To investigate the inhibitory activity of PTBP1 on Y RNA cleavage, recombinant PTBP1 protein which was extracted from 293T cells was incubated with the reaction solution containing Y RNA and R.F. fraction showing RNase activity. Without PTBP1, the majority of Y3 RNA was cleaved into ASRs. In the presence of PTBP1, Y RNA was dramatically protected in dose-dependent manner. The ratio of Y3 RNA/ASRs was more than 8-fold increased. The protective effect was almost completely canceled by the addition of activated caspase 3, indicating that Y RNA cleavage activities were inhibited by PTBP1, and caspase 3 inactivated this PTBP1 inhibition (Fig. 4B). Moreover, the Y RNA cleavage was also examined with “caspase 3-resistant PTBP1” mutant which carries amino acid substitution at caspase 3 target loci (D7A, D139A, and D172A) as described [30]. While wild-type PTBP1 was cleaved by caspase 3, the mutant PTBP1 was uncleaved (Fig. 4C). In this condition, cleavage of Y3 RNA remained inhibited by the mutant PTBP1 even with addition of caspase 3, while that was proceeded by wild-type PTBP1 in a caspase 3 dose-dependent manner. (Fig. 4D). These results demonstrated that the Y RNA cleavage is protected by PTBP1, which is subsequently deactivated by caspase 3 which is responsible for apoptosis-dependent cleavage of Y RNA. (Fig. 4E).

### High positivity of ANA and elevated inflammatory cytokines in *Ptbp1*^*mut/mut*^ mice

**Fig. 5:**
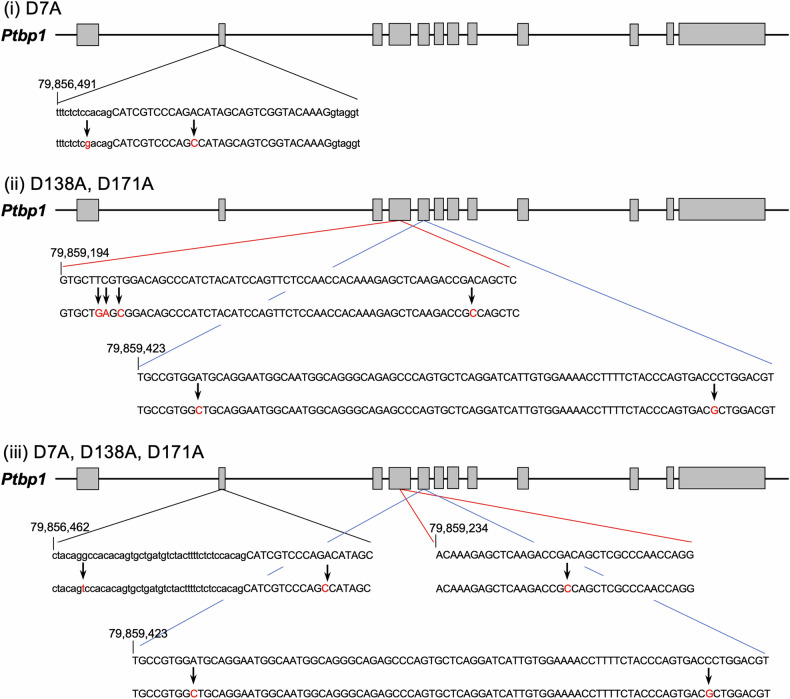
Schematic representation of the genome-edited sequences of *Ptbp1*^*mut/mut*^ mice. The point mutation at (i) D7A, (ii) D138A and D171A in *Ptbp1* gene were introduced into oocyte of C57BL/6 mice by CRISPR/Cas9 system, respectively. Then these mice were inbred to generate (iii) D7A, D138A and D171A triple mutation knock-in mice (Caspase 3-resistant PTBP1 knock-in mice: *Ptbp1*^*mut/mut*^ mice).

**Fig. 6:**
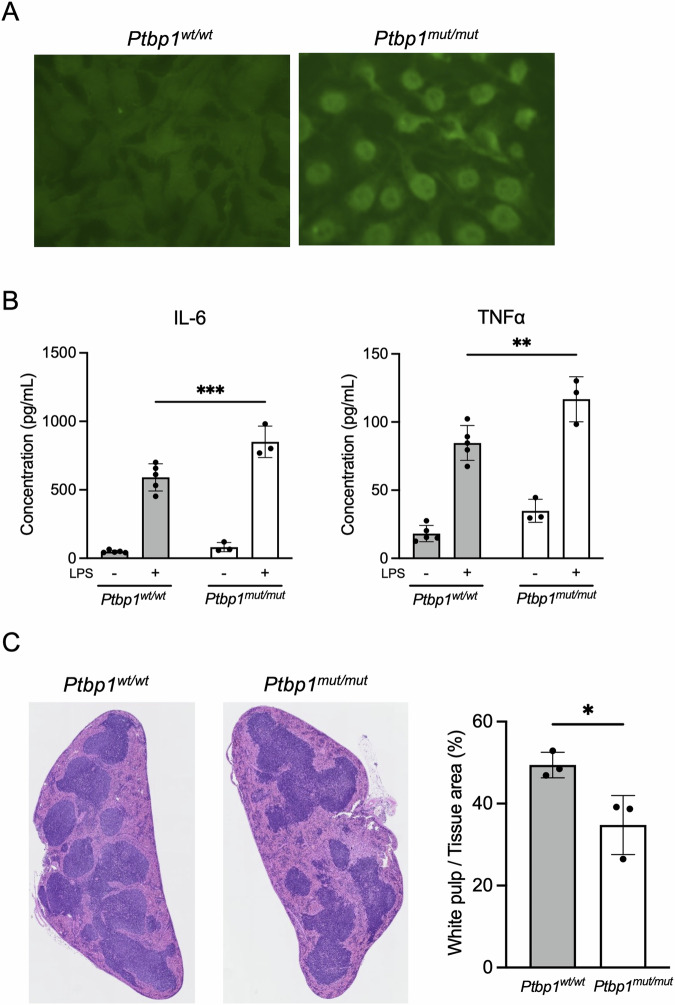
*Ptbp1*^*mut/mut*^ mice show higher positivity of ANA and inflammatory cytokine elevation in the serum. **A** Representative of the indirect immunofluorescence assay on HEp-2 cells performed using the serum of *Ptbp1*^*wt/wt*^ and *Ptbp1*^*mut/mut*^ mice. **B** Results of multiplex cytokine bead assay using the serum of *Ptbp1*^*wt/wt*^ (*n* = 5) and *Ptbp1*^*mut/mut*^ (*n* = 3) mice before and after LPS administration. **C** Representative H&E staining of spleen and the ratio of white pulp to whole tissue area in *Ptbp1*^*wt/wt*^ (*n* = 3) and *Ptbp1*^*mut/mut*^ (*n* = 3) mice after LPS administration. Similar results were obtained in two independent experiments. Student’s t-test was used for the statistical analysis. *P < 0.05, error bars; mean ± s.d.

To elucidate the biological significance of inhibition of Y RNA cleavage by PTBP1 and its cancelation by truncation of PTBP1 by caspase 3, caspase 3-resistant PTBP1 knock-in mice (*Ptbp1*^*mut/mut*^) were established (Fig. 5). We found that these mice were apparently healthy and normal. The *Ptbp1*^*mut/mut*^ mice at 14 weeks exhibited an ANA positivity rate of 16.7%, while their wild-type (*Ptbp1*^*wt/wt*^) littermates showed an ANA positivity rate of 12.5% (data not shown). This suggests that the levels of ANA positivity between the two groups were nearly comparable at this age. However, at 28 weeks old, specifically, *Ptbp1*^*mut/mut*^ mice presented with a 100% (6/6) ANA positivity rate. In comparison, *Ptbp1*^*mut/wt*^ and *Ptbp1*^*wt/wt*^ littermates exhibited positivity rates of 50% (2/4) and 22% (2/9), respectively (Fig. 6A). The IL-6 and TNFa in the serum of the *Ptbp1*^*mut/mut*^ mice were not elevated compared to *Ptbp1*^*wt/wt*^ mice without stimulation (IL-6: *p* = 0.812, TNFa: *p* = 0.120). On the stimulation of LPS, those of the *Ptbp1*^*mut/mut*^ mice were significantly more augmented than those of *Ptbp1*^*wt/wt*^ mice (IL-6: *p* = 0.0010, TNFa: *p* = 0.0038, Fig. 6B). By contrast, *Ptbp1*^*mut/mut*^ mice revealed a marked reduction in the size of the white pulp in the spleen, compared to *Ptbp1*^*wt/wt*^ mice (*p* = 0.032, Fig. 6C). Those results suggest that the deregulation of PTBP1 truncation and/or Y RNA cleavage is involved in immune deregulation and ANA formation.

## Discussion

We previously found ASRs, a class of small non-coding RNAs. Their mechanism of action is similar to that of miRNAs [5]. ASRs were identified in EBV-infected cells and their expression was dramatically elevated during the lytic phase of EBV infection. Recent advancements in next-generation sequencing have revealed the presence of ASRs in various cellular contexts. This suggests that the processing of ASRs might have broader implications, extending beyond EBV infection to encompass general cellular biology functions. In this study, we have found that the precursors of ASRs are non-coding Y RNAs. Y RNAs are evolutionarily conserved and essential for the initiation of DNA replication. Previous study indicates Y RNAs undergo cleavage during apoptosis [9]. The lytic phase of EBV infection is characterized by massive apoptosis, leading to the hypothesis that ASRs emerge from the cleavage of Y RNAs during this phase. Although the small RNA degradation or processing, which includes Y RNAs or U1 snRNA in apoptosis, was previously observed [7, 9], their cleavage enzymes, functions, and biological significance in apoptosis remained unclear.

In this study, we identified PTBP1 as a “Caspase 3-sensitive protector” for Y RNA degradation. As shown in Fig. 1D, Y RNA cleavage was induced by cytoplasmic extracts following treatment with caspase 3. The fractionation of the cell extract using SAS, IEC, and gel-filtration chromatography revealed that at least two molecules, ribonuclease and PTBP1 are involved in the regulation of the machinery. PTBP1 probably binds to the cleavage locus of Y RNA and inhibits the access to RNase (Fig. 4E).

Despite similarities between the ASRs and miRNA machineries for exerting their functions, canonical miRNA-processing enzymes for the biogenesis of miRNAs, DROSHA and DICER, had no role in the processing (Fig. 2A, B). miRNAs are generally processed by DICER, which interacts with AGO protein when miRNA loads onto RNA-induced silencing complex (RISC). We have previously shown that the most distinctive feature of ASRs was to be selectively loaded onto AGO1. It would be interesting to know whether the molecules involved in the processing of ASRs, including unknown RNases, are related to selective AGO1 loading.

We performed mass spectrometry analysis to determine RNase using samples from gel-filtration chromatography and IEC R.F (Fig. 3B, data not shown). However, we failed to identify the molecules, owing to the detection of excess protein species. To overcome this problem, additional fractionation or other screening methods would be needed.

Chromatin fragmentation is a well-characterized event in apoptosis. Caspase 3 activated DNase (CAD) is an endonuclease specifically activated in apoptosis by caspase 3 [31], and CAD lacks a caspase 3 recognition site. On the other hand, an inhibitor protein of CAD (ICAD) has caspase 3 recognition site and usually binds to CAD to prevent DNase activity [3, 32]. On the contrary, PTBP1 originally binds to Y RNA and protects it from RNase, but when PTBP1 is degraded by caspase 3, Y RNA is also degraded. While the degradation of ICAD by caspase 3 promotes DNase activity of CAD, the degradation of PTBP1 by caspase 3 promotes the accessibility of RNase to Y RNA.

Experiments using CAD knockout mice revealed that the systemic deficiency of DNase II, which causes DNA accumulation in macrophages, resulting in excess innate-immune response [33]. In addition, systemic autoimmune diseases such as SjS and SLE are characterized by ANA or ribonucleocomplexes in the patient’s serum. Ro60 and La, which autoantibodies are detected in the serum of autoimmune disease patients, are Y RNA-binding proteins. Although the relationship between the autoantigen and pathogenesis is poorly understood, Y RNA cleavage could have some roles in suppressing the exposure of autoantigens to immunosurveillance [34]. The caspase 3-resistant PTBP1 knock-in mice demonstrated that deregulation of Y RNA cleavage or/and PTBP1 truncation in apoptosis is involved in deregulation of the immune system, possibly causing the generation of autoantigens, leading to the development of autoimmune diseases.

Y RNAs were discovered from the serum of autoimmune disease systemic SLE patients [35]. Human has four Y RNAs genes denoted, Y1, Y3, Y4, and Y5, while mouse has two Y RNAs, which are orthologs of human Y1 and Y3 [36, 37]. In a recent study, human Y5 RNA was cleaved by RNase L in A549 cells, while mouse Y1 RNA was not cleaved by RNase L in MEF cells [38]. Our experimental data showed that mouse Y3 RNA was not cleaved by RNase L (Fig. 2D). Caspase-3-dependent PTBP1 degradation is the trigger for apoptosis mediated degradation of at least Y3-RNA. The RNase which cleaves Y3 RNA should be identified by further investigation.

In this study, we created Caspase 3-resistant *Ptbp1*^*mut/mut*^ mice for the first time. Although PTBP1 is known to be important for RNA metabolism, including splicing, polyadenylation, mRNA translation, localization, and degradation [39], altering the sensitivity of PTBP1 to caspase-3 degradation does not alter development. Instead, we observed the elevated serum cytokines response to LPS stimulation, which seems to contradict with less germinal center formation in *Ptbp1*^*mut/mut*^ mice, but suggests immune dysregulation in those mice. Interestingly, PTBP1 is necessary for the B cell selection in germinal centers and controls mRNA abundance and alternative splicing in these cells [40]. In addition, caspase 3-mediated apoptosis is crucial in germinal center homeostasis [41]. However, further investigation is needed to elucidate the detailed molecular mechanism.

In summary, we investigated the molecular machinery of apoptotic Y RNA cleavage. Y RNAs are protected by its inhibitor PTBP1, and caspase 3 induces the truncation of PTBP1 and apoptotic Y RNA cleavage by a cytoplasmic endoribonuclease, which is involved in the generation of ANA.

## Materials and methods

### Cells

Epstein-Barr virus-positive Akata cells and Jurkat cells were maintained in RPMI-1640 medium (Wako) supplemented with 10% (v/v) fetal bovine serum (FBS), 50 U/mL penicillin, and 50 μg/mL streptomycin. Dicer- or RnaseL- (kindly gifted by Dr. R. Silverman) deficient mouse embryonic fibroblasts (MEFs) and HEK293T cells were maintained in DMEM (Nacalai Tesque) supplemented with 10% (v/v) FBS, 50 U/mL penicillin, and 50 μg/mL streptomycin.

### Apoptosis and lytic phase induction

To induce apoptosis, MEF cells and Jarkat cells were treated with 10 μM staurosporine (Wako) for 2–6 h and anti-Fas antibody (clone: CH-11) for 6 h, respectively.

To induce the EBV lytic phase, Akata cells were stimulated with rabbit anti-human IgG polyclonal antibody (20 mg/mL) (Dako) at 37 °C for 24 h. Apoptosis was detected by propidium iodide (PI)/allophycocyanin (APC)-Annexin V (Merck and BD Bioscience) staining and analyzed by FACS Verse (BD Bioscience).

### Northern blotting

The purified total RNA (5 μg) was mixed with an equal volume of gel loading buffer II (Ambion) and denatured at 95 °C for 5 min. Denatured samples were separated by electrophoresis using 15% urea gel and transferred onto Hybond N+ membrane (GE Healthcare) in the cold room. Hybridization was performed with ULTRAhyb™ buffer (Ambion) at 37 °C for overnight. Digoxigenin (DIG)-labeled Locked Nucleic Acid (LNA) probe for ASRs derived from Y3 RNA was purchased from EXIQON. Hybridized probes were detected by anti-DIG-AP, Fab fragments, and CSPD substrate (Roche). The detection probe for ASRs derived from Y5 RNA and U6 RNA was prepared using the DIG oligonucleotide tailing kit, 2nd generation (Roche), according to the manufacturer’s instructions.

### Transfection with siRNAs

We purchased siRNAs targeting human DROSHA and DICER from OriGene Technologies. siRNase L, siPTBP1, and siY RNA were obtained from Hokkaido System Science. AccuTarget^TM^ Negative Control siRNA (Bioneer) was used as control. Transfection was performed with the Neon transfection system (Invitrogen) according to the manufacturer’s protocol. PTBP1 knockdown was detected using the mean fluorescent intensity (MFI) of fused EGFP, with FACS Verse. Other target genes were evaluated by real-time PCR. Sequences of specific siRNAs are described in Supplemental Table S1.

### Quantification of gene expression

Total RNA was purified from cells, using Sepasol-RNA I Super G (Nacalai Tesque), and reverse transcribed using the High-Capacity cDNA transcription kit (Thermo Fisher Scientific). Real-time PCR was performed using THUNDERBIRD SYBR qPCR Mix (TOYOBO) with StepOnePlus real-time PCR system (Applied Biosystems). Threshold cycle (Ct) values were calibrated to β-actin and analyzed by the 2^−⊿⊿CT^ method. Gene-specific primers are described in Supplemental Table S1.

### Protein fractionation and ribonuclease activity assay

Jurkat cells were suspended in an equal volume of a buffer containing 50 mM PIPES-KOH (pH 7.4), 50 mM KCl, 5 mM EGTA, 2 mM MgCl_2_, 1 mM DTT, 20 µM cytochalasin B (Wako), and protease inhibitor cocktail (Merck). The suspension was immediately frozen in ice-cold isopropanol bath and melted on ice. The thawed sample was disrupted by the Dounce homogenizer (KONTES) with 50 strokes. The freeze, thaw, and disrupt cycle was repeated. The lysate was centrifuged at 10,000 × *g* for 12 min at 4 °C and moved to a new tube. The supernatant was separated by ultracentrifugation at 100,000 × *g* and 4 °C for 90 min. The cytoplasmic fraction (S-100) of Jurkat cells was separated by ultracentrifugation and treated with ^32^P-labeled Y3 RNA for 2 h at 30 °C with or without 0.25 unit of activated caspase 3. RNAs were purified from the reaction and separated by urea-PAGE. S-100 fraction was separated by ammonium sulfate fractionation. The collected supernatant was subjected to ion-exchange chromatography (IEC) with HiTrap Q column (GE healthcare) and eluted by a linear gradient of 0-500 mM NaCl. ^32^P-labeled Y3 RNA was prepared using an in vitro transcription T7 kit (TaKaRa). Each fraction and ^32^P-labeled Y3 RNA were mixed in 10 mM HEPES-KOH pH 7.0, 50 mM NaCl, 2 mM MgCl_2_, 20% glycerol, 40 mM beta-glycerophosphate, 5 mM DTT, and 1 mg/mL bovine serum albumin (BSA) and incubated with or without caspase 3 (kindly gifted by Dr. S. Nagata) for 2 h at 30 °C. RNA was purified from the reactions by acid-phenol and ethanol precipitation and separated by 15% acrylamide-denatured gel (urea). The gel was exposed to an imaging plate and analyzed by FLA-2000 (FUJIFILM).

### Western blotting

Each sample was separated by sodium dodecyl sulfate-polyacrylamide gel electrophoresis (SDS-PAGE). The separated proteins were transferred onto a polyvinylidene difluoride (PVDF) membrane (Merck) and the membrane was blocked using a PVDF blocking reagent for Can Get Signal (TOYOBO) at 25 °C for 1 h. Blotting was performed with anti-PTBP1 antibody (RN011P, MBL). Horseradish peroxidase (HRP)-conjugated secondary antibody or streptavidin was added to the membranes and the peroxidase activity was detected using Immobilon western chemiluminescent HRP substrate (Merck).

### Recombinant PTBP1 preparation

We cloned PTBP1 into pcDNA3-Flag and transfected the vector into 2 × 10^7^ HEK293T cells, using polyethylenimine “MAX” (Polysciences). After 2 days of transfection, the nuclear lysate was mixed with anti-FLAG M2 affinity gel (Merck) and eluted by incubation with 3× Flag peptide (Merck). A cDNA of StrepTagII-tagged PTBP1 was cloned into pET49 plasmid. To generate the vector expressing caspase 3-resistant PTBP1, site-directed mutagenesis was performed using Pfu Turbo DNA Polymerase (Agilent Technologies), with three primer pairs carrying the nucleotides for D7A, D139A, and D172A mutations. These vectors were transformed into *Escherichia coli* BL21(DE3) strain. The lysates were injected into StrepTrap HP column (GE Healthcare) and eluted with 2.5 mM dethtiobiotin (Merck).

### Generation of *Ptbp1*^*mut/mut*^ mice

Oligonucleotide covering the partial genome of *Ptbp1* with D7A mutation and that with D138A and D171A mutations are inserted using CRISPR/Cas9 system to oocytes of C57BL/6 mice [42]. Then the mice which have D7A mutation (D7A mutant knock-in mice) and those that have D138A and D171A mutations (D138A /D171A mutant knock-in mice) were inbred to generate triple mutation knock-in mice (Caspase 3-resistant *Ptbp1* knock-in mice: *Ptbp1*^*mut/mut*^ mice). Each *Ptbp1* mutation was confirmed by PCR and direct sequencing. Sequences of each *Ptbp1* mutation are described in Supplemental Table S2.

### Antinuclear antibody detection assay

Blood samples were collected from the facial veins of mice (26–28 weeks). Serum antinuclear antibodies (ANA) were detected as previously described using a 1:10 dilution of serum on HEp-2 ANA slides as an initial screen. Bound antibody was detected with goat anti-human IgG FITC (MBL). Slides were observed using an IX81 microscope (Olympus Life Science).

### Cytokine analysis

Serum levels of IL-6 and TNFa were determined using Cytometric Bead Array Mouse IL-6 Flex Set and TNFa Flex Set (BD Biosciences) according to the manufacturer’s instructions. All samples were read on a FACS Lyric (BD Biosciences).

### Immunohistochemistry

Twenty hours after intraperitoneal administration of LPS (3 mg/kg), the blood and spleen were collected from mice (38–44 weeks). Spleen specimens were fixed in 10% formalin and embedded in paraffin. Spleen specimens were cut from the tissue block in 4-μm sections and stained with hematoxylin and eosin. The ratio of white pulp to whole tissue area was determined with Image J software.

### Statistical analysis

Statistical significance was obtained using two-sided Student’s *t*-test and set at *p*-value < 0.05. All experiments were independently replicated. Specific sample sizes and the count of independent experiments conducted for each study can be found in the figure legends.

### Supplementary information

Supplemental Table S1

Supplemental Table S2

Original data files

## Data Availability

Correspondence and request for materials should be addressed to AK.
