# Supplementary figures and images for: PTBP1 protects Y RNA from cleavage leading to its apoptosis-specific degradation
Source: Cell Death Discov. 2024 Jul 12;10:322. doi: 10.1038/s41420-024-02080-6 (PMC11245482; doi:10.1038/s41420-024-02080-6)

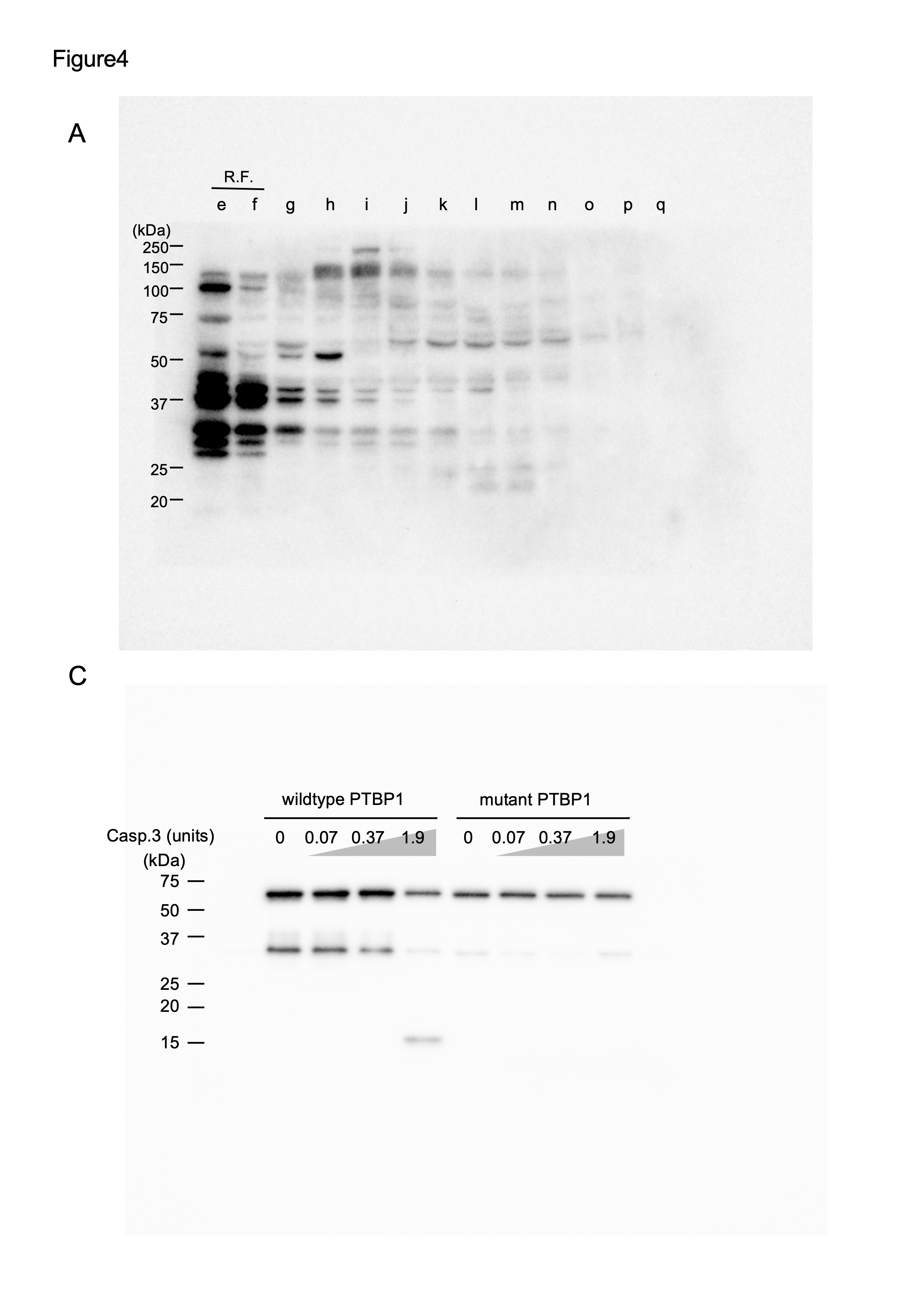

Supplement: Supplementary file 3 — Original data files [file 41420_2024_2080_MOESM3_ESM.tif]
